# YOLO-B:An infrared target detection algorithm based on bi-fusion and efficient decoupled

**DOI:** 10.1371/journal.pone.0298677

**Published:** 2024-03-28

**Authors:** Yanli Hou, Bohua Tang, Zhen Ma, Juan Wang, Ben Liang, Yongqiang Zhang

**Affiliations:** 1 School of Information Science and Engineering, Hebei University of Science and Technology, Shijiazhuang, Hebei, PR China; 2 Hebei Technology Innovation Center of Intelligent IoT, Shijiazhuang, Hebei, PR China; Purdue University, UNITED STATES

## Abstract

The YOLO-B infrared target detection algorithm is proposed to address the problems of incomplete extraction of detailed features and missed and wrong detection of infrared targets by YOLOv5s. The algorithm improves the SPPF of YOLOv5s feature extraction network by proposing the CSPPF structure to increase the sensory field of the model. The Bifusion Neck structure is invoked to fuse the shallow location information with deep semantic information to enhance the feature extraction capability of the model. Taking fully into account the different information of concern for classification and localization, the efficient decoupled head is used as the prediction head of this algorithm, which reduces the latency while maintaining the accuracy. WIoUv3 loss is used as a bounding box regression loss function to reduce the harmful gradient generated by low-quality examples and reduce the competitiveness of high-quality anchor frames. Comparative experiments were conducted for each of the four improvement points, and the experimental results showed that each improvement point had the highest detection accuracy in the comparative experiments of the same category. All improvement points are fused in turn and ablation experiments are performed. The YOLO-B algorithm improves 1.9% in accuracy, 7.3% in recall, 3.8% in map_0.5, and 4.6% in map_0.5:0.95 compared to YOLOv5s. When compared with YOLOv7 and YOLOv8s, the proposed algorithm has better performance in terms of the number of parameters and detection accuracy.

## Introduction

The combination of infrared thermal imaging technology and target detection solves the shortcomings of visible images that do not work continuously throughout the day, and overcomes the impact of light on detection. Due to its significant research and application value, Infrared target detection technology has garnered considerable attention in the field of computer vision [1–4]. Despite the remarkable achievements of existing target detection algorithms, most of them are for visible images and still face many problems in infrared image detection.

Currently, there are two primary classifications for target detection algorithms based on deep learning: two-stage and single-stage. The two-stage algorithm first generates the candidate region of interest on the image and then performs classification regression, which has high detection accuracy, but is computationally intensive and slow in detection, represented by R-CNN [5], Fast-RCNN [6], Faster-RCNN [7], Mask R-CNN [8], etc. The single-stage detection algorithm integrates the target frame prediction and recognition into one step, and completes the target localization and classification regression directly on the image through the preset anchor frame [9], which avoids the step of generating candidate regions, effectively reduces the network computation and greatly improves the detection speed, and the representative algorithms are: YOLO series [10–15], SSD [16], etc. A lot of researchs have been done in the field of infrared target detection based on these algorithms. Zhao et al. [17] proposed a multiscale infrared target detection method based on bi-directional feature fusion, and proposed a bi-directional feature fusion structure called Improve-FPN (Im-FPN) to achieve fast and efficient multiscale feature fusion, and used the Focal Loss (FL) loss function to balance the problem of sample imbalance. Based on these improvements, the detection ability of the model has been enhanced compared to YOLOv3, but the detection accuracy is low compared to similar algorithms. Sun et al. [18] constructed a model I-YOLO for road infrared target detection based on YOLOv3 by introducing an expanded residual structure U-Net, and the experimental results show that the model not only achieves high detection accuracy but also maintains high detection speed. Li et al. [19] proposed a small target detection method for infrared remote sensing images based on super-resolution and YOLOv5, which effectively solved the problems of missed detection and false alarms by adding an attention mechanism and introducing Swin Transformer Blocks to replace the bottleneck layer of the C3 module in the network head. However, the above two studies lacked the description of the number of model parameters and the computational amount. Zhao et al. [20] proposed a novel multi-scale target detection method for infrared vehicle targets based on YOLOv7, which introduces a lightweight MobileViT network to reduce the complexity of the model. An innovative C3-PANet neural network structure was designed to improve the model’s recognition accuracy of the target area. Experiments on the HIT-UAV public dataset show that the proposed algorithm improves the mAP by 0.9% over the existing algorithm, which proves the effectiveness of the improved algorithm, but the number of parameters of the improved model is high, which is not conducive to mobile deployment.

Although the aforementioned infrared target detection algorithms have achieved many notable results, these algorithms still suffer from low detection accuracy and high model complexity. In order to simultaneously take into account the detection accuracy and model complexity, the improved YOLO-B infrared target detection algorithm based on YOLOv5s is proposed. In the YOLO-B algorithm, for the characteristics of poor contrast and difficulty in extracting detail information in infrared images, the CSPPF and Bifusion Neck structures are designed to enhance the ability of extracting detail information in infrared images, and to improve the model detection accuracy. Specific innovations are listed below:

1. The CSPPF structure is proposed to increase the sensory field of the network model and avoid the loss of detailed information during pooling.
2. Bifusion Neck is employed to strengthen the feature extraction capability of the model, to fuse the shallow detail information and deep semantic information, and to improve model detection accuracy.
3. The efficient decoupled head is used as the prediction head of the proposed algorithm to alleviate the extra delay overhead caused by 3 × 3 convolution, improve the misalignment problem of classification and prediction regression, and solve the computationally intensive problem of YOLOv5s coupling head while maintaining accuracy.
4. The WIoUv3 loss is employed for the purpose of bounding box regression, with the aim of diminishing the competitiveness posed by anchor boxes of superior quality and mitigating the detrimental gradients arising from subpar examples, ultimately elevating the detector’s overall performance.

## Related work

The existing infrared target detection algorithms are classified into two major categories based on the detection principles, one is the traditional infrared target detection method based on manually designed and the other is the infrared target detection method based on deep learning.

The conventional approach to feature extraction, which relies on manual, possesses certain limitations. Traditional infrared target detection algorithms usually use shallow information such as grayscale values, edges, and textures to acquire the position and class of the target, and cannot adequately extract semantic information for deeper layers. The generalization ability of the model is poor when facing scenes such as complex backgrounds and dense occlusion of targets.

The emergence of deep learning algorithms for object detection has solved the difficulties encountered by traditional algorithms, and the feature extraction capability as well as the model generalization capability have been significantly improved.

Xue et al. [21] proposed a real-time pedestrian detection method with multimodal attention fusion YOLO to efficiently detect pedestrians at night. The algorithm improved the robustness of the model in detecting small targets by constructing a multimodal feature extraction module, weighting and fusing the modal features, and acquiring the semantic features of small targets by introducing a double attention module. Wu et al. [22] introduced an algorithm with U-shaped structure named ISTDU-Net, which was designed for detecting infrared small targets. The algorithm enhanced the weight of small targets by introducing feature map group perception, while incorporating a fully connected layer into the jump connection to enhance the discernibility between the target and the background. Gao et al. [23] proposed an anchor-free lightweight infrared target detection method, which introduced the attention mechanism and channel shuffle, and strengthened the characterization of the lightweight network through the SkipBranch structure, so that the deep feature information and shallow feature information were fully integrated. Li et al. [24] proposed an image generation algorithm for infrared ship-enhanced StyleGAN. By introducing a self-attention mechanism, it focused on texture detail information as well as pixel correlation information, utilized a wavelet discriminator to scale down the generation of artifacts, and introduced a WGAN-gp loss function to enhance the convergence efficiency. The quality of the enhanced infrared ship images is significantly improved. Zhang et al. [25] proposed an anti-jamming recognition algorithm for DNET aerial infrared image targets based on information feature extraction. The algorithm used a dense connection module for large-scale feature images, introduced a feature attention mechanism at the end of the network, and added a multi-scale dense connection module in the multi-scale fusion part. The improved algorithm had high recognition accuracy and recall, fast recognition speed, and good robustness in the case of infrared decoy interference. Chen et al. [26] presented an attention-based encoder-decoder convolutional neural network (AED-CNN). The network enabled the interaction of features between different scales by introducing an encoder-decoder module, eliminated the background information and enhanced the pedestrian information by re-weighting the encoder-decoder module with the introduction of an attention module, and significantly improved the detection accuracy on publicly available datasets. Ju et al. [27] proposed an efficient end-to-end network, ISTDet, which utilized an image filtering module to obtain a confidence map and an infrared small target detection module to discriminate the class and position of the targets. ISTDet was trained in an end-to-end manner, aiming to enhance the detection performance.

## Methods

**Fig 1:**
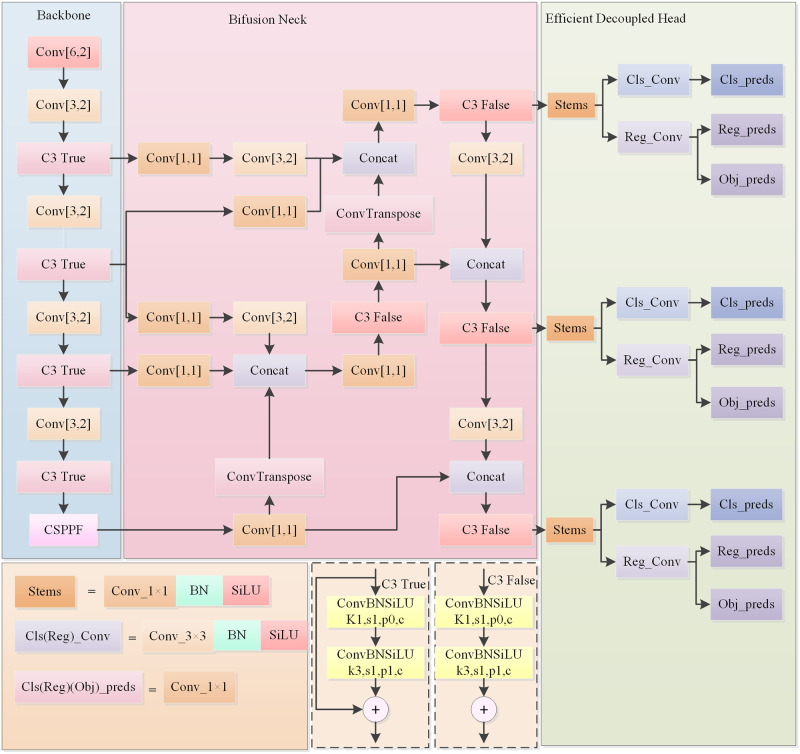
YOLO-B structure.

YOLO-B uses YOLOv5s as the baseline model, focusing on improving the neck of the network as well as the detection head. Firstly, the SPPF of the backbone network is improved to obtain the new structure CSPPF. Secondly, Bifusion Neck is introduced to replace the PANet of YOLOv5s, and the detection part uses the efficient decoupled head so that the classification task and the regression task are performed separately. Finally, the newly proposed WIoUv3 loss is used as a regression loss function to improve the overall performance of the detector. The structure of YOLO-B is shown in Fig 1.

### CSPPF

SPPF (Spatial Pyramid Pooling-Fast) passes the inputs in parallel through maximum pooling layers of different sizes and then does further fusion. SPPF can address the problem of multi-scale targets to a certain extent and enable the network to realize adaptive sized outputs.

**Fig 2:**
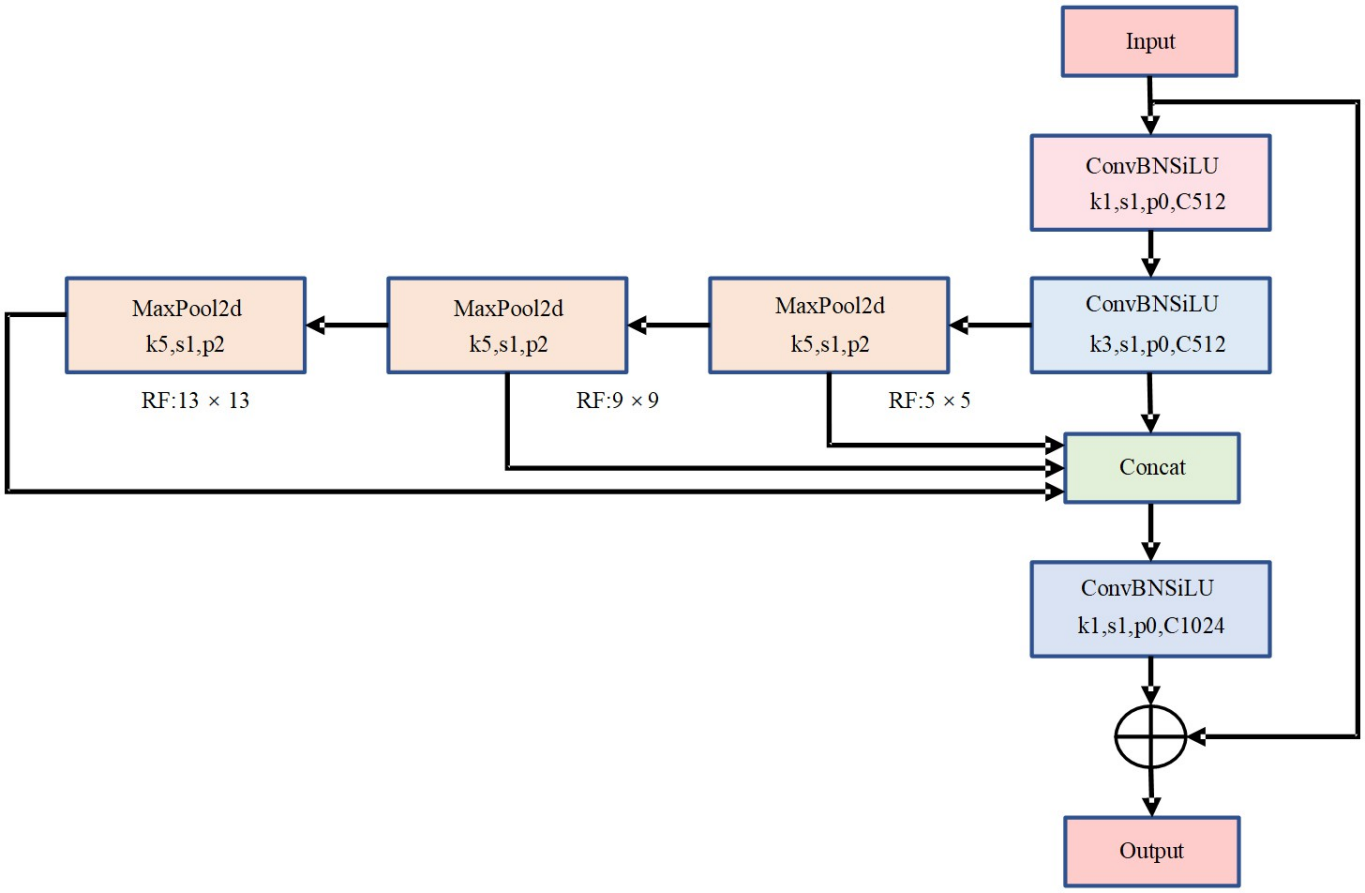
CSPPF structure.

The SPPF will lose the detail information of the target after multiple pooling, and the loss of edge detail information will lead to the accuracy decreasing [28, 29]. For this problem, the SPPF is improved to get a new structure CSPPF, whose structure is shown in Fig 2. The CSPPF module branches the input features, one branch is shorted, the other branch undergoes 1 × 1 convolution for dimensionality reduction, and then feature extraction is performed by 3 × 3 convolution. The extracted features are serially passed through multiple maximal pooling layers, and the output of each pooling is used as the input for the next pooling, and then the output of the pooling layers are fused with the multi-scale features. The dimensionality reduction of the fused features add the first shortcut branch, which can retain the original input information as much as possible to compensate for the information lost in the pooling process.

### Bifusion Neck

YOLOv5s uses PANet (Path Aggregation Network, PANet) [30] as the feature fusion network. PANet adds an extra bottom-up path to FPN, through which it enhances the feature hierarchy at the top of the feature pyramid network, shortens the information paths of the bottom and top features, and helps to propagate accurate signals from the low-level features, but the bidirectional fusion of PANet is simpler, and it is challenging to cope with infrared target detection in complex backgrounds.

Target identification by learning scale features is the key to locating targets. In order to fully and efficiently exchange multi-scale information, a new structure Bifusion Neck is obtained by improving PANet. Bifusion Neck integrates the features of the three nearest-neighbor layers of the backbone output with a bidirectional cascade (BiC) module, and this processing preserves more accurate localization information.

**Fig 3:**
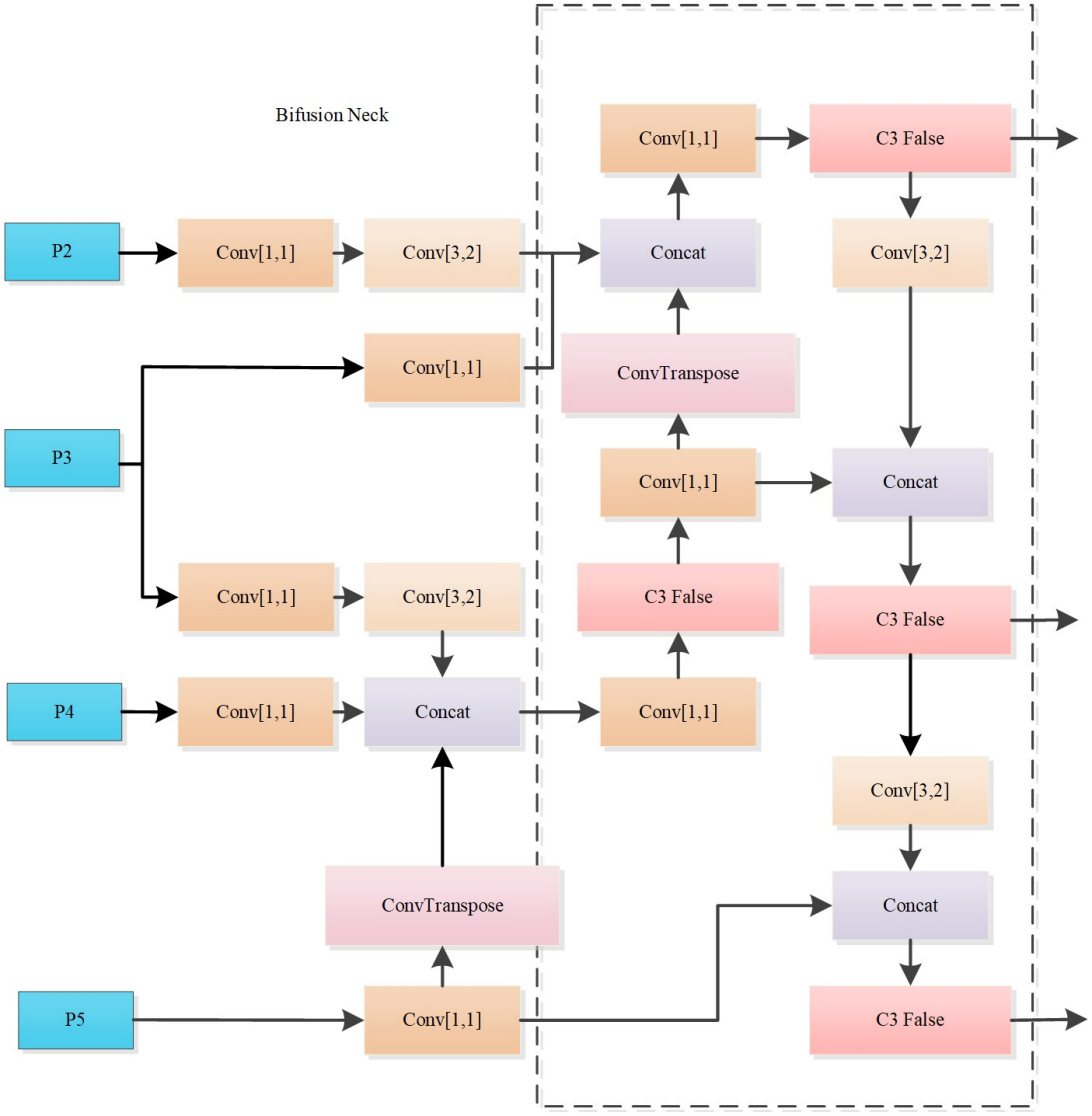
Bifusion Neck structure.

The specific structure of Bifusion Neck is shown in Fig 3, and its improvement can be divided into three main parts. Firstly, Bifusion Neck has an additional output of P2 feature layer compared to PANet, and P2, as a lower feature layer, retains more shallow feature information. Secondly, the input feature maps are reduced in dimensionality using 1 × 1 convolution to reduce the computational effort of the model. Thirdly, the large-scale feature map P3 is downsampled by Conv(3,2), the small-scale feature map P5 is upsampled by ConvTranspose, and then they are concatenated with the medium-scale feature map P4, which realizes the full fusion of the low-level features with the high-level features, and makes the localization information more accurate.

### Efficient decoupled head

The detection head of YOLOv5s realizes the detection task by fusion sharing of classification and regression, i.e., both classification and regression are done in a single 1 × 1 convolution. However, classification and regression focus on different regions of interest, as classification is responsible for finding the closest of the existing categories for the extracted features, while localization is responsible for focusing on the location coordinates and thus for parameter correction of the bounding box. So YOLOv5s detection head classifies and locates in a feature map at the same time, which is prone to localization misalignment problems.

**Fig 4:**
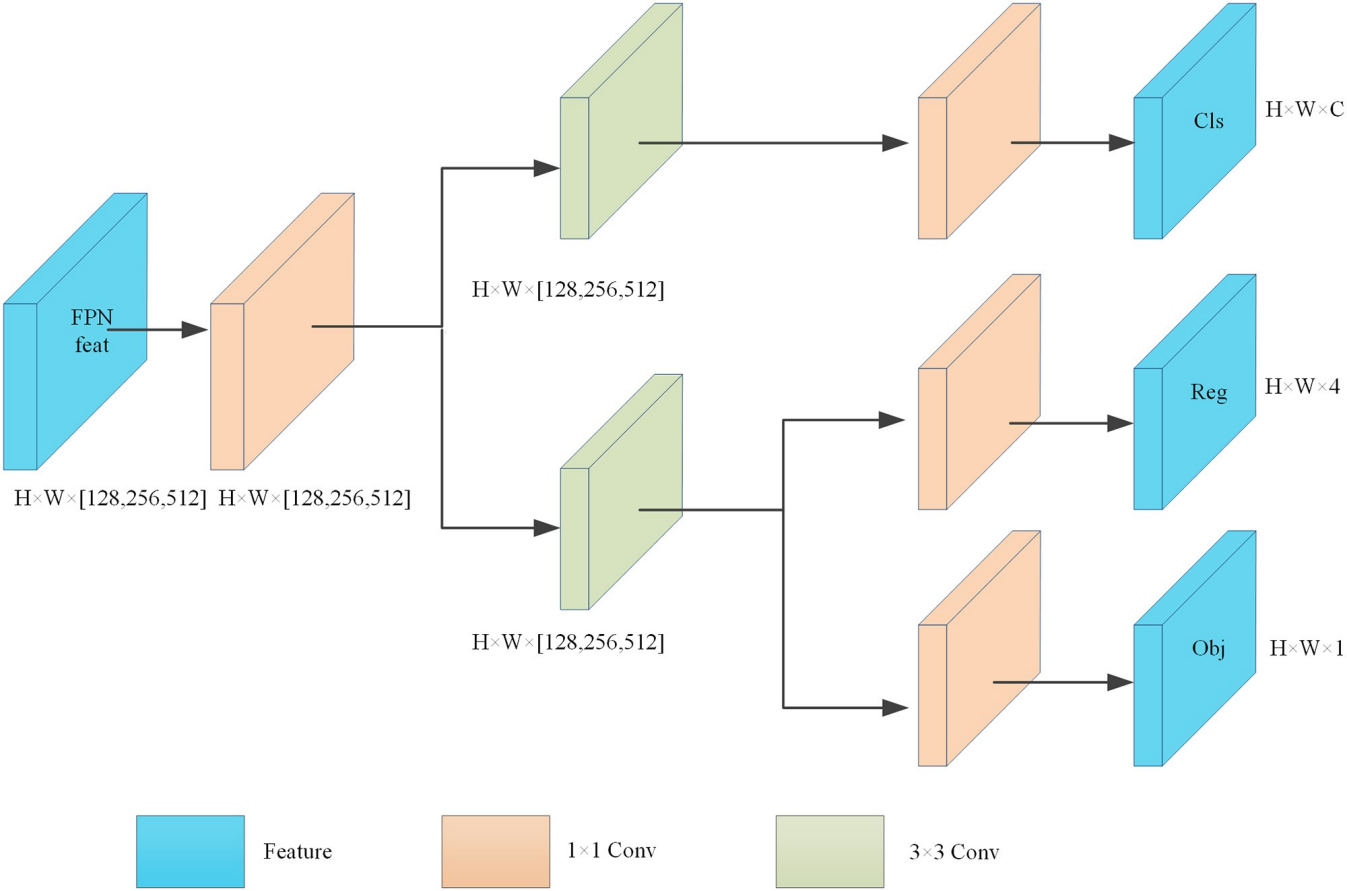
Efficient decoupled head structure.

To address the above problem, the classification and regression tasks are implemented separately by invoking the efficient decoupled head in YOLOv6. The efficient decoupled head is borrowed from decoupled head design of the YOLOX [31] and improved. The efficient decoupled head is shown in Fig 4. Compared with the YOLOX decoupled head, the efficient decoupled head not only alleviates the extra delay overhead caused by 3 × 3 convolution, but also improves the misalignment problem of classification and prediction regression, and reduces the delay while maintaining the accuracy and solving the computationally intensive problem of the YOLOX decoupled head.

### WIoUv3 loss

In the YOLOv5s algorithm, the CIoU loss [32] is used as the bounding box regression localization loss function. The CIOU loss function fails to account for the true disparity between aspect ratios and confidence levels, thus hindering the model from optimizing similarity effectively, resulting in inaccurate localization.

In order to improve the localization performance of the model, distance attention is constructed based on the distance metric, and WIoUv1 [33] with two-layer attention mechanism is obtained. The loss function of the WIoUv1 is shown in Eqs 1 and 2.

$$\begin{array}{c} L_{WIOUv1}=R_{WIOU}L_{IOU} \end{array}$$ (1)

$$\begin{array}{c} R_{WIOU}=exp\frac{{\left(x-x_{gt}\right)}^{2}+{\left(y-y_{gt}\right)}^{2}}{{\left(W_{g}^{2}+H_{g}^{2}\right)}^{*}} \end{array}$$ (2)

Where *R*_*WIOU*_ ∈ [1, *e*) indicates the penalty item, *L*_*IOU*_ ∈ [0, 1] indicates the IoU loss, *W*_*g*_ and *H*_*g*_ are the size of the smallest enclosing box.

The optimization is continued on the basis of WIoUv1 by introducing *β* to construct non-monotonic focusing coefficients to obtain the WIoUv3 [34] loss function. The loss function of the WIoUv3 is shown in Eqs 3 and 4.

$$\begin{array}{c} L_{WIOUv3}=rL_{WIOUV1} \end{array}$$ (3)

$$\begin{array}{c} r=\frac{\beta }{\delta \alpha^{\beta -\delta }} \end{array}$$ (4)

Where *r* denotes the gradient gain, *β* denotes the outlier degree, *α* and *δ* are hyperparameters. The outlier degree is employed to depict the caliber of the anchor box, wherein the diminished outlier degree connotes heightened anchor box quality.

To ensure that low-quality anchor boxes do not lag behind during the initial stages of training, we assign the highest gradient gain to anchor boxes with an IOU loss of 1. During the later stages of training, WIoUv3 allocates small gradient gains to low-quality anchor boxes in order to mitigate harmful gradients. Meanwhile WIoUv3 will focus on anchor boxes of ordinary quality, enhancing the positioning performance of the model.

## Materials

### Dataset

This experiment uses the FLIR public infrared dataset [34], which contains 14,452 thermal images, and 8,862 images remaining after removing invalid data, containing a total of 67,618 targets. The dataset includes a variety of conditions such as daytime, nighttime, foggy days, rainy days, etc. The image background includes mountains, tunnels, buildings, etc., and the background is relatively complex. The main labeled categories of the dataset are person, car, and bicycle, and the training set add validation set, and test set are divided in a randomized manner, adhering to the proportion of 8:2. The dataset labels distribution are shown in Table 1.

**Table 1:** FLIR dataset label distribution.

Class	Train	Val	Test	Total
Person	14249	3639	4484	22372
Car	26574	6449	8237	41260
Bicycle	2604	555	827	3986
Total	43427	10643	13548	67618

**Fig 5:**
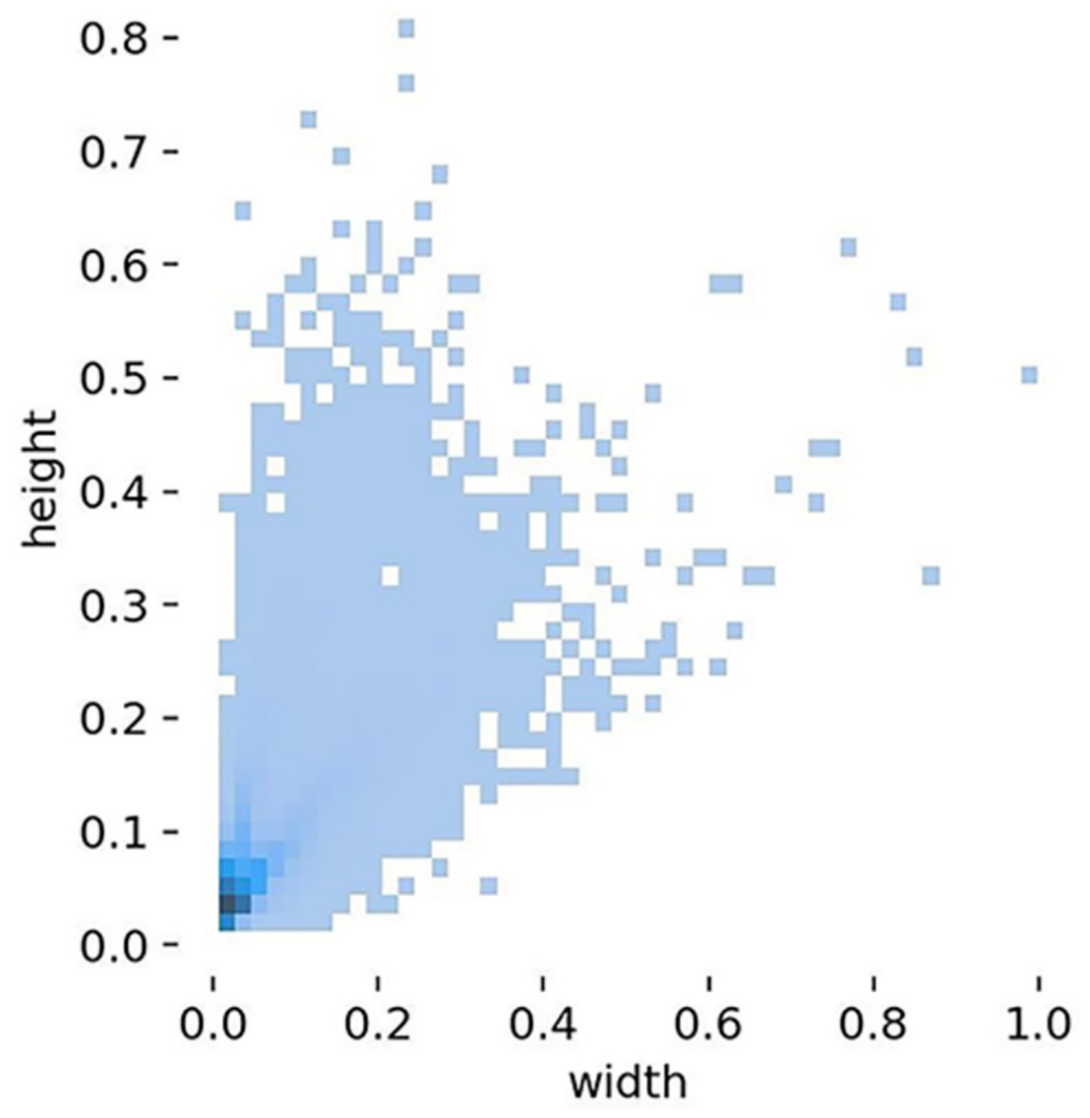
Distribution of labels aspect ratio.

As can be seen from the distribution of label aspect ratios in the dataset shown in Fig 5, small-sized targets are predominant in the dataset, making detection more difficult.

The KAIST [35] pedestrian dataset consists of a total of 95,328 images, the train set contains 50,187 images, and the test set contains 45,141 images. The dataset captures various regular traffic scenarios including campus, street, and countryside during daytime and nighttime respectively. The label of the dataset contains the classes person, people and cyclist. Since the KAIST dataset is poorly labeled and neighboring images do not differ much, some degree of data cleaning was performed and all classes were uniformly labeled as person. After data cleaning, we can obtain 7601 train set images and 2252 test set images. The specific distribution of the KAIST dataset is shown in Table 2.

**Table 2:** Distribution of the KAIST dataset.

Class	Train	Test	Total
Day	4755	1455	6210
Night	2846	797	3643
Total	7601	2252	9853

### Experimental environment

The experimental platform uses Intel(R) XeonW-2245 CPU with NVIDIARTX 3080 GPU, 10G video memory and 32GB memory size. In Windows 10 OS, Pytorch1.7 framework is built, CUDA version is 11.2, and the training process is accelerated with GPU.

The initial learning rate of all experimental models is 0.01, the gradient momentum is 0.937, and the weight decay coefficient is 0.0005. The learning rate is warmed up with epoch of 3 and momentum parameter of 0.8, and the learning rate is updated using cosine annealing after warming up. The training batchsize is 16, and the total number of training rounds is 300 epochs.

### Evaluation metrics

The model performance is evaluated using Precision (P), Recall (R), mean Average Precision (mAP), and F1-score (F1). The calculation formulas are shown in Eqs 5, 6, 7 and 8.

$$\begin{array}{c} P=\frac{TP}{TP+FP} \end{array}$$ (5)

$$\begin{array}{c} R=\frac{TP}{TP+FN} \end{array}$$ (6)

$$\begin{array}{c} mAP=\frac{1}{n}\sum_{i=1}^{n}\;AP_{i} \end{array}$$ (7)

$$\begin{array}{c} F_{1}=\frac{2PR}{P+R} \end{array}$$ (8)

Where *AP*_*i*_ represents the accuracy rate of the ith category, *TP* represents the number of samples correctly classified as positive, *FP* represents the number of samples incorrectly classified as positive, *FN* represents the number of samples incorrectly classified as negative, *F*_*n*_ represents the number of images detected, and *T* represents the total time taken for detection.

## Results and discussion

The experimental mainly includes the following parts: module comparison experiments, ablation experiments and comparison experiments with different algorithms. Module comparison tests and ablation experiments are performed on FLIR dataset to verify the effectiveness of the improved module. Conduct experiments on FLIR dataset and KAIST dataset respectively to compare with the latest target detection algorithms to demonstrate the effectiveness and generalization of the YOLO-B algorithm.

### Module comparison experiment results

In order to explore the effectiveness of the improved modules, the proposed modules were compared in experiments based on YOLOv5s, and Precision, Recall, mAP, parameters and GFLOPS were selected as evaluation metrics for this experiment, and the same data set, hyperparameter settings and training techniques were used in each group.

#### Comparative experiments of CSPPF

The CSPPF is applied to the YOLOv5s detector and compared experimentally with SPPF, SimSPPF [14], SPPCSPC [15], ASPP [36], and BasicRFB [37]. The findings from the comparative experimentation are displayed within Table 3.

**Table 3:** Comparative experimental results of CSPPF.

Module	Precision	Recall	mAP0.5	mAP0.5:0.95	GFLOPS	Parameters(M)
CSPPF	0.856	0.795	0.857	0.472	16.2	7.60
SPPF	0.843	0.744	0.830	0.443	15.8	7.01
SimSPPF	0.864	0.776	0.846	0.453	15.8	7.01
ASPP	0.860	0.766	0.841	0.452	22.4	15.20
BasicRFB	0.860	0.775	0.847	0.459	16.3	7.67
SPPCSPC	0.867	0.776	0.852	0.464	20.9	13.44

As evidenced by the data presented in Table 3, CSPPF achieves the highest Recall and mAP in comparison with the other five modules. In terms of parameters and GFLOPs, CSPPF is almost the same as SPPF, SimSPPF, and BasicRFB, and significantly lower than ASPP and SPCSPC. Therefore CSPPF has the best performance compared to other modules.

#### Comparative experiment of Bifusion Neck

The Bifusion Neck is applied to the YOLOv5s detector and compared experimentally with FPN [38], PANet [30], BiFPN [39], and Slim-Neck [40], and the outcomes of the comparative experiments are presented in Table 4.

**Table 4:** Comparative experimental results of Bifusion Neck.

Module	Precision	Recall	mAP0.5	mAP0.5:0.95	GFLOPS	Parameters(M)
Bifusion Neck	0.861	0.796	0.859	0.473	16.0	7.10
FPN	0.837	0.738	0.825	0.439	16.0	6.85
PANet	0.843	0.744	0.830	0.443	15.8	7.01
BiFPN	0.877	0.756	0.843	0.451	16.4	7.16
Slim-Neck	0.856	0.770	0.840	0.447	15.1	7.93

The experimental findings illustrated in Table 4. showcase that the five Neck models, FPN, PANet, BiFPN, Slim-Neck, and Bifusion Neck, have low parameters and GFLOPs, and do not rely on high-performance servers for training. So the five modules focus on three types of metrics, namely recall, precision, and mAP, when comparing them. Bifusion Neck achieves the highest value in recall, precision, and mAP compared to the other four Necks, and applying it to the YOLOv5s model, the detection performance can be optimized.

#### Comparative experiment of Efficient decoupled head

The efficient decoupled head is applied to the YOLOv5s detector and compared experimentally with the coupled head in YOLOv5s and the decoupled head in YOLOX, and the findings from the comparative experimentation are displayed within Table 5.

**Table 5:** Comparative experimental results of Efficient decoupled head.

Module	Precision	Recall	mAP0.5	mAP0.5:0.95	GFLOPS	Parameters(M)
Coupled head	0.843	0.744	0.830	0.443	15.8	7.01
Decoupled head	0.861	0.800	0.857	0.477	56.2	15.32
Efficient decoupled head	0.854	0.794	0.854	0.474	22.0	8.92

As evidenced by the data presented in Table 5, the efficient decoupled head increases the parameters by 1.91M over the coupled head, mAP_0.5 improves by 1.2%, and mAP_0.5 decreases by 0.3% over the decoupled head, but the parameters and GFLOPs is only half of that of the decoupled head. From the combination of model complexity and detection accuracy, the efficient decoupled head has the best detection performance among the three detection heads.

#### Comparative experiments of WIoUv3 loss

The WIoUv3 loss is applied to the YOLOv5s detector and compared experimentally with CIoU loss [32], EIoU loss [41], and SIoU loss [42], and the experimental comparison results are shown in Table 6.

**Table 6:** Comparative experimental results of WIoUv3 loss.

Module	Precision	Recall	mAP0.5	mAP0.5:0.95
CIoU loss	0.843	0.744	0.830	0.443
EIoU loss	0.842	0.790	0.853	0.456
SIoU loss	0.873	0.777	0.851	0.457
WIoUv3 loss	0.860	0.788	0.855	0.459

The four loss functions in Table 6 have the same number of parameters and GFLOPS, so these two evaluation metrics are not considered in this experiment. Precision, Recall and mAP of WIoUv3 loss is the highest among the five loss functions, and the comprehensive performance is optimal.

**Fig 6:**
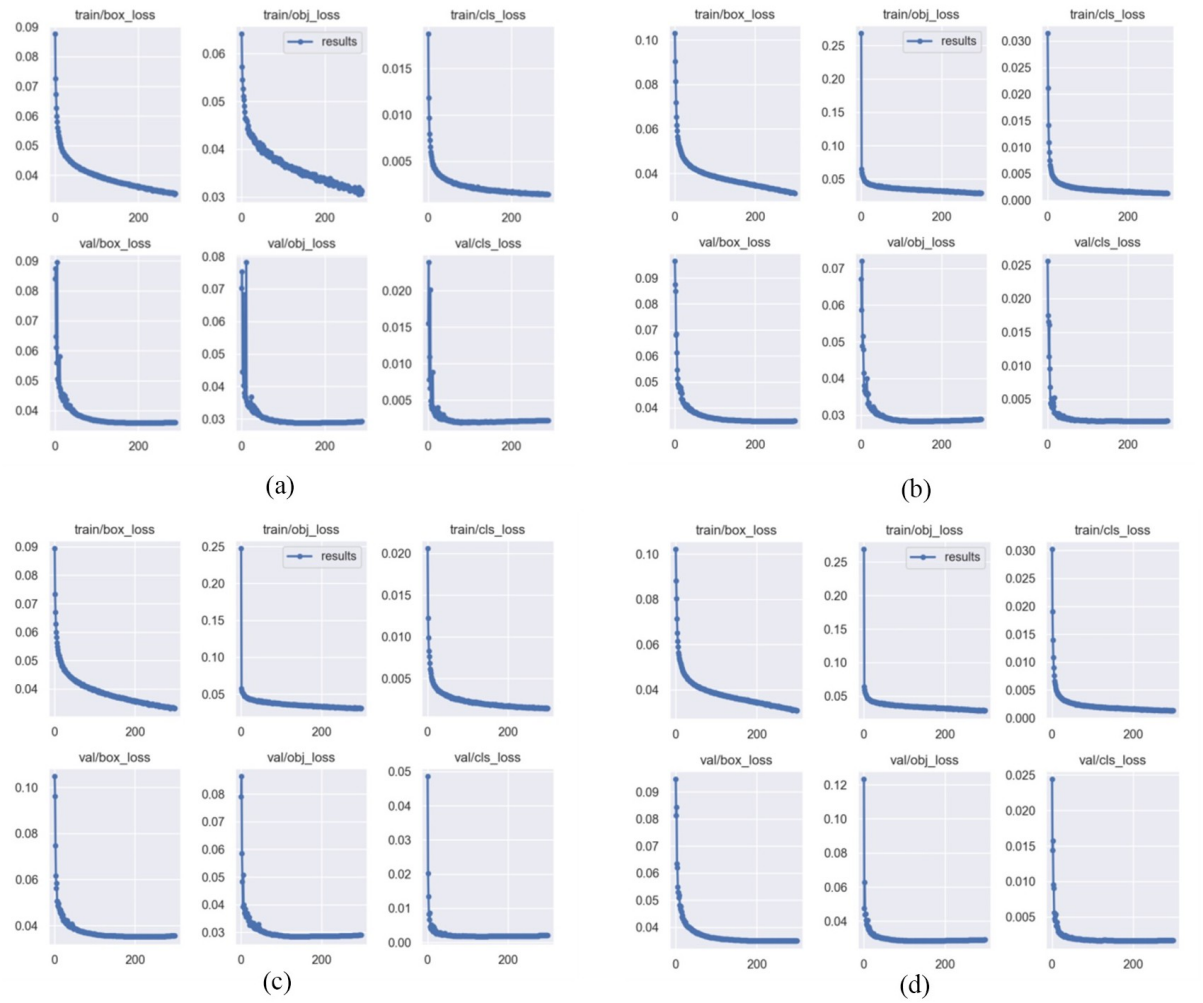
Loss curves of the four loss functions. a: CIoU loss. b: EIoU loss. c: EIoU loss. d: SIoU loss.

In order to gain a more intuitive understanding of the impact of the loss function on the model, Fig 6 presents the loss curves of four different loss functions for both the training and validation datasets.

As can be seen from Fig 6, the loss curves of the WIoUv3 loss converge the fastest and have the flattest curves. In summary, WIoUv3 loss is applied to the YOLOv5s model, which improves the model’s learning and generalization abilities, and provides the best detection among the four loss functions.

### Ablation experiment results

Ablation experiments were performed on the FLIR dataset to authenticate the efficacy of each module. The experimental findings are demonstrated as depicted in Table 7, indicated by ✔ for using the improved method and ✘ for not using the improvement. Due to limited space on the table, ‘Efficient decoupled head’ will be abbreviated as ‘EDH’.

**Table 7:** Ablation experiment results.

CSPPF	Bifusion Neck	EDH	WIoUv3	Precision	Recall	mAP0.5	mAP0.5:0.95	Parameters(M)
✘	✘	✘	✘	0.843	0.744	0.830	0.443	7.01
✔	✘	✘	✘	0.856	0.795	0.857	0.472	7.60
✔	✔	✘	✘	0.859	0.810	0.860	0.476	7.76
✔	✔	✔	✘	0.861	0.812	0.865	0.480	8.92
✔	✔	✔	✔	0.862	0.817	0.868	0.489	8.92

Based on the findings depicted in Table 7, it is evident that the recall rate is improved by 5.1%, mAP_0.5 by 2.7% and mAP_0.5:0.95 by 2.9% adding the CSPPF module, which can retain the original input information, compensate for the information lost in the pooling process and improve the ability of the model to focus on features. The addition of the Bifusion Neck feature fusion structure improved mAP_0.5 and mAP_0.5:0.95 by 0.3% and 0.4%, respectively. Bifusion Neck enhances the model’s ability to recognize small and dense targets and improves the model’s generalization capability. The use of the efficient decoupled head alleviates the extra delay overhead and improves the misalignment between classification and predictive regression, with an improvement of 0.5% for mAP_0.5 and 0.4% for mAP_0.5:0.95. Finally, the utilization of the WIoUv3 loss function enhances the convergence rate of the model, with an increase of 0.3% and 0.9% observed for mAP_0.5 and mAP_0.5:0.95 correspondingly. All the improved modules were fused, and the final improved model YOLO-B Precision reached 86.2%, Recall reached 81.7%, mAP_0.5 reached 86.8%, and mAP_0.5:0.95 reached 48.9%. mAP_0.5 and mAP_0.5:0.95 values compared to YOLOv5s were increased by 3.8% and 4.6%.

### Comparative experiment results of different algorithms

#### Comparison experiments on the FLIR dataset

The YOLO-B algorithm is experimentally compared with nine current mainstream algorithms. The comparison experiments is divided into two groups, one for the lightweight algorithm and the other for the algorithm with a larger number of parameters. Lightweight algorithms with smaller number of parameters, GFLOPs and model size, and are easier to develop and deploy on hardware devices to fulfill the need for real-time detection. The algorithms chosen for comparative analysis include the lightweight YOLOv3-tiny, YOLOv5s, YOLOv5-ghost, YOLOv5-repvgg, and YOLOv7-tiny. The experimental results can be found in Table 8.

**Table 8:** Comparative experiments of lightweight algorithms.

Method	Precision	Recall	mAP0.5	Parameters(M)	GFLOPs	Model size(MB)
YOLOv3-tiny	0.816	0.636	0.722	8.67	12.9	17.4
YOLOv5s	0.843	0.744	0.830	7.01	15.8	13.7
YOLOv5-ghost	0.848	0.744	0.827	3.68	8.10	7.90
YOLOv5-repvgg	0.861	0.794	0.855	7.01	15.8	14.8
YOLOv7-tiny	0.829	0.770	0.842	6.01	13.0	12.3
YOLO-B	0.862	0.817	0.868	8.92	22.0	15.8

As evidenced by the depiction Table 8, YOLO-B algorithm has a 2.1MB increase in model size, 1.91M increase in number of parameters, and 6.2G increase in FLOPs compared to YOLOv5s. However, the precision rate P and recall rate R reached 86.2% and 81.7%, which were significantly improved compared with YOLOv5s, effectively reducing the model false detection rate, and the mAP_0.5 improved by 3.8 percentage points to 86.8%, improving the model detection accuracy. Accuracy, recall and mAP are also far ahead compared to YOLOv3-tiny, YOLOv5-ghost, YOLOv5-repvgg and YOLOv7-tiny lightweight models.

Meanwhile, so as to further verify the universality and generalization of the YOLO-B, we compared it with the YOLOv5m, YOLOv6s, YOLOv7 and YOLOv8s algorithms with larger number of parameters. The experimental results can be found in Table 9.

**Table 9:** Comparative experiments of large size algorithms.

Method	Precision	Recall	mAP0.5	Parameters(M)	GFLOPs	Model size(MB)
YOLOv5m	0.855	0.796	0.859	20.86	47.9	42.2
YOLOv6s	0.857	0.810	0.864	17.2	44.2	36.3
YOLOv7	0.864	0.818	0.872	36.49	103.2	74.8
YOLOv8s	0.859	0.799	0.865	11.12	28.4	22.5
YOLO-B	0.862	0.817	0.868	8.92	22.0	15.8

As evidenced by the depiction Table 9, YOLO-B reaches the minimum value in the second group of experiments in terms of the number of model parameters, computational effort, and model size. The YOLO-B algorithm mAP_0.5 is 86.8%, which is 0.9% higher compared to YOLOv5m, 0.4% and 0.3% higher than YOLOv6s and YOLOv8s respectively, and just 0.4% lower compared to YOLOv7, but with 27.57MB less number of params than YOLOv7.

#### Comparison experiments on the KAIST dataset

In order to further verify the effectiveness and generalization of YOLO-B algorithm, the performance of YOLO-B algorithm is compared with the latest target detection algorithms on KAIST dataset. The comparison experiments are divided into two groups, the first group selects the lightweight algorithms YOLOv5s, YOLOv6n, and YOLOv7-tiny. The second group selects YOLOv5m, YOLOv6s, YOLOv7, YOLOv8s with larger number of parameters. The experimental results are shown in Table 10.

**Table 10:** Comparison experiments on the KAIST dataset.

Method	Precision	Recall	mAP0.5	Parameters(M)	GFLOPs	Model size(MB)
YOLOv5s	0.576	0.485	0.513	7.01	15.8	13.7
YOLOv6n	0.558	0.467	0.472	4.63	11.3	9.95
YOLOv7-tiny	0.573	0.515	0.488	6.01	13.0	12.3
YOLO-B	0.597	0.489	0.523	8.92	22.0	15.8
YOLOv5m	0.588	0.488	0.522	20.86	47.9	42.2
YOLOv6s	0.576	0.508	0.485	17.2	44.2	36.3
YOLOv7	0.591	0.501	0.493	36.49	103.2	74.8
YOLOv8s	0.585	0.504	0.512	11.12	28.4	22.5

As can be seen from Table 10, in the first set of comparison experiments, YOLO-B achieved the highest P, R, and mAP. Compared to YOLOv5s, P increased by 2.1%, R increased by 0.4%, and mAP_0.5 increased by 1%. In the second set of comparison experiments, YOLO-B achieves the highest P and mAP, and at the same time achieves the smallest values in three aspects: the number of model parameters, the amount of computation, and the model size. According to the two sets of comparison experiments, it can be seen that YOLO-B has taken both model size and model accuracy into account, and achieved a significant improvement in detection accuracy at the cost of a small increase in model complexity.

The YOLO-B algorithm achieves excellent detection results on both the FLIR dataset and the KAIST dataset, which fully proves the effectiveness and generalizability of YOLO-B. However, YOLO-B still has some problems, firstly, the dataset used in the paper contains fewer target categories, which leads to poorer generalization ability of the model and limits the applicability of the model in practical applications. Secondly, the number of parameters, GFLOPS, and model size of YOLO-B have increased compared to baseline.

Although the algorithm proposed in this paper has room for improvement, YOLO-B is undeniably an excellent infrared target detection algorithm.

## Conclusion

The YOLO-B-based infrared target detection algorithm is proposed for the problems of inconspicuous infrared target texture detail features and difficult feature extraction. The YOLO-B algorithm uses YOLOv5s as the baseline to improve the SPPF of the backbone network, and proposes a new CSPPF structure. By introducing the Bifusion Neck and the efficient decoupled head to optimize the Neck and Head sections. Ultimately, the adoption of the WIoUv3 loss function enhances the model’s learning capabilities and generalization prowess. The results of the ablation experiment indicate that compared with the YOLOv5s algorithm, the YOLO-B algorithm improves mAP_0.5 by 3.8% and mAP_0.5:0.95 by 4.6% with only 1.91M increase in the number of parameters, and generates more accurate target detection frames. The YOLO-B algorithm is tested against nine mainstream algorithms, and the experimental results show that the YOLO-B algorithm has better performance in terms of both detection accuracy and model computation, with stronger expressiveness as well as generalization ability and better robustness.

In future work, we will expand the dataset to add more categories of targets including small targets, do further lightweighting of the model and integrate thermal matching with our algorithm [43, 44].

## Supporting information

S1 Dataset All information for this experimental dataset is stored at https://github.com/Tbhkkl/dataset. (DOCX)
