# Supplementary material for: YOLO-B:An infrared target detection algorithm based on bi-fusion and efficient decoupled
Source: PLoS One. 2024 Mar 28;19(3):e0298677. doi: 10.1371/journal.pone.0298677 (PMC10977756; doi:10.1371/journal.pone.0298677)
Supplement: S1 Dataset — (DOCX) [file pone.0298677.s001.docx]

**Supporting information:** S1 Dataset. All information for this experimental dataset is stored at https://github.com/Tbhkkl/dataset.
